# Differential activation of human neutrophils by SARS-CoV-2 variants of concern

**DOI:** 10.3389/fimmu.2022.1010140

**Published:** 2022-10-27

**Authors:** Samuel Lebourgeois, Ambroise David, Houssem Redha Chenane, Vanessa Granger, Reyene Menidjel, Nadhira Fidouh, Benoît Noël, Olivier Delelis, Clémence Richetta, Charlotte Charpentier, Sylvie Chollet-Martin, Diane Descamps, Benoit Visseaux, Luc de Chaisemartin

**Affiliations:** ^1^ Université Paris Cité, Infection Antimicrobials Modelling Evolution (IAME), Institut National de la Santé et de la Recherche Médicale (INSERM), Paris, France; ^2^ Assistance Publique - Hôpitaux de Paris (AP-HP), University Hospital Bichat-Claude Bernard, Laboratoire d’Immunologie, Paris, France; ^3^ Inflammation, Microbiome and Immunosurveillance, Université Paris-Saclay, Institut National de la Santé et de la Recherche Médicale (INSERM), Châtenay-Malabry, France; ^4^ Assistance Publique - Hôpitaux de Paris (AP-HP), University Hospital Bichat-Claude Bernard, Laboratoire de Virologie, Paris, France; ^5^ LBPA-Laboratoire Biologie Pharmacologie Appliquée, Ecole Normal Supérieur (ENS) Paris-Saclay, Centre National de la Recherche Scientifique (CNRS) Unité Mix de Recherche (UMR), Université Paris-Saclay, Gif-sur-yvette, France

**Keywords:** human neutrophils, SARS-CoV-2, variant of concerns, netosis, IL-8

## Abstract

The emerging SARS-CoV-2 virus has affected the entire world with over 600 million confirmed cases and 6.5 million deaths as of September 2022. Since the beginning of the pandemic, several variants of SARS-CoV-2 have emerged, with different infectivity and virulence. Several studies suggest an important role of neutrophils in SARS-Cov-2 infection severity, but data about direct activation of neutrophils by the virus is scarce. Here, we studied the *in vitro* activation of human neutrophils by SARS-CoV-2 variants of concern (VOCs). In our work, we show that upon stimulation with SARS-Cov-2 infectious particles, human healthy resting neutrophils upregulate activation markers, degranulate IL-8, produce Reactive Oxygen Species and release Neutrophil Extracellular Traps. Neutrophil activation was dependent on TLR7/8 and IRF3/STING. We then compared the activation potential of neutrophils by SARS-CoV-2 variants and showed a significantly increased activation by the Delta variant and a decreased activation by the Omicron variant as compared to the initial strain. In this study, we demonstrate that the SARS-Cov-2 virus can directly activate neutrophils in COVID-19 and that the different VOCs had differences in neutrophil activation intensity that mirror the differences of clinical severity. These data highlight the need to address neutrophil-virus interactions as a potential target for therapeutic intervention in SARS-CoV-2 infection.

## Introduction

The emerging SARS-CoV-2 virus was first identified in Wuhan City, China, on 31 December 2019 as the etiological agent of coronavirus infectious disease 2019 (COVID-19). It was subsequently declared pandemic by the World Health Organization (WHO) on 11 March 2020 (1). Until 15 September, 2022, the entire world has been affected by this virus with over 600 million confirmed cases and over 6.5 million deaths.

Since the beginning of the pandemic, several variants of SARS-CoV-2 have emerged. The first successful emergence was observed between March and April 2020 with the spread of the D614G mutation. This mutation was associated with higher viral loads and improved adhesion to the cellular angiotensin-converting enzyme 2 (ACE2) receptor (2). Since the late 2020s, several new variants of concern (VOC) have been identified, including the Alpha (B.1.1.7), Beta (B.1.351), Gamma (P.1) and then Delta (B.1.617.2) variants, which have rapidly and widely dominated in Europe and worldwide. More recently, the Omicron variant (B.1.1.526) has come to the forefront of global concern. However, this variant, which is thought to have a much higher adhesion capacity than other VOCs (3), seem to induce a less severe disease (4).

In humans, coronavirus infection generally causes mild respiratory infections such as common cold, including fever, cough and shortness of breath (5). Several clinical profiles have been described, but none of them was associated to a specific risk factor (6). Most people infected are only mildly symptomatic, but approximately 20% of patients may progress to severe disease with a risk of acute respiratory distress syndrome (ARDS), sepsis, multiorgan dysfunction and ultimately death (5). The overactivation of the innate immune response against SARS-CoV-2, including production of pro-inflammatory cytokines (TNFα, IL-6, IL-1β and IFN-γ/α) is thought to play a major role in the pathophysiology (7).

Among the first immune cells to be recruited and activated in infections are neutrophils. Neutrophils possess formidable anti-infectious weaponry including secretion of proteases and cytokines, production of reactive oxygen species (ROS), and even expulsion of extracellular DNA filaments called neutrophil extracellular traps, or NETs (8). Although neutrophils are more studied in bacterial or fungal infections, they are now recognized to be of importance during viral infections as well (8, 9). In COVID-19 infection, increased circulating neutrophil numbers and neutrophil recruitment to lungs have been described and linked to severity (10). Additionally, elevated NETs have been measured in blood and tissues and associated to lung injury, thrombosis, and severity (11, 12).

However, despite the large amount of data available on the activation status of neutrophils in COVID-19, there is still little information on how the neutrophils respond to the SARS-CoV-2 itself, and particularly to the different VOCs of the virus. In this study, we explored the neutrophil activation potential of all the major VOCs of SARS-CoV-2 *in vitro*, and demonstrated a significant disparity in neutrophil activation intensity depending on the variant.

## Material and methods

### Viral strains

The viral strains of human SARS-CoV-2 VOCs were obtained from a positive nasopharyngeal PCR sample. The viruses have been treated in biosafety level-3 laboratory (BSL-3). The SARS-CoV-2 primo-culture stocks used as B (EPI_ISL_4537783), Alpha (EPI_ISL_4536454 and EPI_ISL_4536996), Beta (EPI_ISL_4537125 and EPI_ISL_4537284), Gamma (EPI_ISL_4536760), Delta (EPI_ISL_4536228) and Omicron (EPI_ISL_13017139) were produced in Vero E6 cells. The infected Vero E6 cells were incubated at 37°C in a humidified atmosphere with 5% of CO2. The supernatants were purified and concentrated in Amicon^®^Ultra-15 diafiltration devices (Merck Milipore Ltd). Then, supernatants were quantified for viral RNA levels by RT-qPCR (Altona ^®^, Roche) and viral infectivity by lysis plaque assay titration (13) (see below). Supernatants were then aliquoted and stored at −80°C until use. All sequences of culture strains have been sequenced with Nanopore technology (Agilent technologies^®^) using the Artic protocol.

### Viral titration

SARS-CoV-2 infectious titers were obtained by a lysis-plaque assay as previously described (13). Briefly, Vero E6 cells were seeded onto a 12-well plate at a density of 100,000 in DMEM with 10% FBS. The next day, cells were infected by 10 to 10 serial viral dilutions with the same infection protocol than for our viral infection assays (13). After the viral adsorption period of 1 h at 37°C, 500 µl of an agarose medium mix was added. After 3-day incubation at 37°C with 5% of CO2, the supernatant was removed and cells were fixed with 1 ml of a 6% formalin solution for 30 min. The formalin solution was then removed, and cells were colored with a 10% crystal violet solution for 15 min. All wells were then washed with distilled water and dried on bench-coat paper.

### SARS-CoV-2 pseudovirus assay

To compare the differences in neutrophil activation as a function of SARS-CoV-2 VOCs, lentiviral particles pseudotyped with spike proteins encoded for B, Delta and Omicron have been used. We used a NLENG1-ES-IRES plasmid coding for a derived HIV-1 virus NL4-3 (14). The virus produced by this plasmid are all defective carrying two stop codons in the reading frame of the envelope and expressing a fluorescent reporter gene GFP. Also, we used spike SARS-CoV-2 and VOCs expression plasmid (B, Delta and Omicron)(pLV-Spike, *In vivo*gen). Briefly, pseudoviruses were produced in HEK293T cells by lipofectamine co-transfection of NLENG1-ES-IRES and pLV-Spike plasmids. The virus production have been concentrated and purified using a 100kDa specific filter (Amicon^®^, Milipore). Viruses’ productions were stored at -80°C until use. A measure of the viral load was performed on all purified viruses suspensions as previously described (15).

### Neutrophil surface activation markers

Heparinized whole blood neutrophils were counted on an automated hematometer (Sysmex) and the blood was adjusted to a concentration of 4x10^6^ millions neutrophils/mL. Alternatively, neutrophils were magnetically isolated (see below). Neutrophils were incubated with virus at multiplicity of infection (MOI) ranging from 0.1 to 500 or medium for 90 min at 37°C, CO_2_, H_2_0. Anti-CD62L and anti-CD11b fluorescent antibodies (Becton-Dickinson) were added for 15 min at 4°C. Whole blood samples were then subjected to red blood cell (RBC) lysis (BD FACS Lysing Solution, Becton-Dickinson). After washing, cells were acquired on a FACS Lyrics cytometer (Becton-Dickinson). Results were expressed as percentage of activated CD62L^low^/CD11b^bright^ neutrophils. When indicated, whole blood was pre-incubated for 30 min with 10µM cytochalasin D (Sigma), 50nM TLR7/8 inhibitor ODN 2088 (Miltenyi), or 1µM IRF-3 inhibitor BX795 (*In vivo*gen).

### IL-8 measurement

Degranulated IL-8 was measured in supernatants from virus-activated neutrophils after 90 min of contact (short incubation to avoid measuring neosynthesized cytokines) using Human IL-8 Duoset ELISA kit (Bio-techne) according to manufacturers’ recommendations.

### ROS measurement

Heparinized whole blood was pre-incubated with 600ng/ml dihydroethidium (DHE, Sigma-Aldrich) ROS probe for 15 min at 37°C under agitation in a water bath before adding the virus at MOI 0.1-500 or medium for 45min at 37°C, CO_2_, H_2_0. Samples were then subjected to RBC lysis and washing before acquisition on a FACS Lyrics cytometer. Results were expressed in mean fluorescence intensity (MFI) of the probe.

### Apoptosis measurement

Apoptosis was measured on virus-treated neutrophils at MOI 100 using Apoptosis staining kit I (BD Biosciences) according to manufacturer’s recommendations. Alternatively, neutrophils were fixed and permeabilized using Cytofix/Cytoperm kit (BD Biosciences) according to manufacturer’s recommendations, and stained for 30 min with anti-activated Caspase-3 antibody (Clone C92-605 BD Pharmingen).

### NETosis assay

Neutrophils were purified from EDTA-treated whole blood using MACSXpress Neutrophil isolation kit (Miltenyi Biotech) which allowed a purity routinely over 98%. Purified neutrophils were suspended at 1x10^6^ cells/mL and seeded in 12-well plates. Virus at MOI 100 or medium was added and the plates were incubated at 37°C, CO_2_, H_2_0 for 3h. The supernatant was then collected and DNA-MPO complexes were measured by an in-house ELISA as previously described (16). Additionally, DNA-MPO complexes were measured in sera from COVID-infected patients using the same in-house method.

### Patients

Whole blood and neutrophils used for *in vitro* stimulation experiments were collected from healthy volunteers from Etablissement Français du Sang. Samples from 35 SARS-Cov-2 infected patients used to measure NETs were collected upon admission to Bichat Hospital in the context of a clinical study approved by an Ethics committee with consent of patients (National Ethics committee “Ile de France 8”n°2020-A02676-33).

### Statistics

Comparison between paired data set was done with paired Wilcoxon signed-rank test or Friedman test for comparison of more than two groups. Comparison of unpaired data sets was done with Mann-Whitney test or Kruskal-Wallis test for comparison of more than two groups followed by Dunn post-test. Analyses were done in Graphpad Prism v9.3.1 (GraphPad Software, LLC.). A p-value <0.05 was considered significant.

## Results

### Neutrophil activation by SARS-CoV-2 virus requires actin polymerization, TRL7/8 and IRF-3

**Figure 1:**
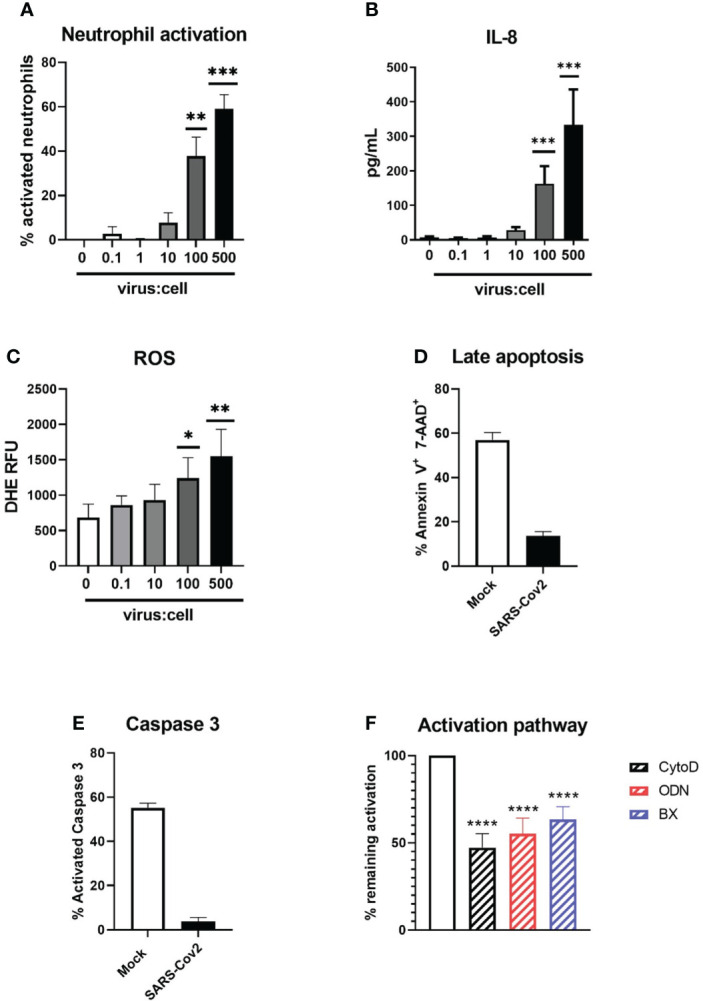
Activation of neutrophils by SARS-CoV-2 infectious particles. **(A)** Percentage of CD11bhigh/CD62Llow activated neutrophils after 90 min incubation of whole blood with SARS-CoV-2 (“Wuhan” B. strain)(n=12). **(B)** Concentration of IL-8 in supernatant after incubation with SARS-CoV-2 (n=12). **(C)** ROS production of neutrophils after incubation with SARS-CoV-2 virus. RFU, relative fluorescence units (n=12). **(D, E)** Percentage of (d) annexin V+/ 7-AAD+ late apoptotic neutrophils and (e) neutrophil expressing active caspase 3 after 18h of incubation of SARS-CoV-2 virus or medium (Mock)(n=12). **(F)** Percentage of inhibition of neutrophil activation by actin polymerization inhibitor cytochalasin D, TLR7 and TLR8 inhibitor ODN 2088 and STING/TLR3 signalling inhibitor BX795 (n=12). Data are mean ± SEM, *p<0.05; **p<0.01; ***p<0.001, ****p<0.0001.

A significant increase in CD11b^hi^/CD62L^low^ activated neutrophils from whole blood could be seen after 90 min of incubation at relatively high MOI (100 to 500, p=0.0002 for both compared to medium) with the ancestral Wuhan strain (B.) of SARS-CoV-2 (**Figure 1A**). A similar activation could be observed on magnetically isolated neutrophils at the same MOIs (**Figure S1**). To confirm this activation, we additionally measured degranulated IL-8 (**Figure 1B**) and ROS production (**Figure 1C**). Both IL-8 and ROS were significantly increased in virus-treated neutrophil supernatants as compared to medium at MOI 500 (333 ± 102 *vs* 8.2 ± 1.9pg/mL, p=0.0001; and 1551 ± 155 *vs* 683 ± 78 relative fluorescence units (RFU), p=0.0012, respectively). After 18h of incubation, late apoptotic cells were dramatically reduced in neutrophils incubated with SARS-CoV-2 compared to the medium alone (13.6% *vs* 57.0%, p<0.0001) (**Figure 1D**), in line with a strongly reduced active caspase 3 expression (3% *vs* 55%, P=0.0022) indicating that SARS-CoV-2 is able to delay neutrophil spontaneous apoptosis (**Figure 1E**).

To decipher neutrophil activation pathways, neutrophils were preincubated with several inhibitors prior to stimulation with the virus (**Figure 1F**). Neutrophil activation could be effectively reduced by inhibition of actin polymerization (52.7 ± 7.9% inhibition, p<0.0001), suggesting endocytosis is necessary for virus-induced neutrophil activation. In line with this, we found that neutrophil activation could also be reduced by inhibition of endosomal RNA-sensing pattern recognition receptors (PRR) TLR7/8 (45% ± 9, p<0.0001). Intriguingly, inhibition of IRF-3, a transcription factor downstream of nucleic acid sensors TLR3 and STING, could also significantly inhibit neutrophil activation (36.7% ± 7, p<0.0001). Since neutrophils do not possess TLR3, this suggests that the cytoplasmic receptor STING could also participate in neutrophil activation. Similarly, RIG-I and MDA-5, known to recognize viral double-stranded RNA, could also induce activation of IRF3. However, this would need cytoplasmic replication of SARS-Cov2 in neutrophils, which has not been observed so far.

Thus, our data show that human neutrophils can be directly activated by SARS-CoV-2 virus in an actin- and PRR-dependent manner.

### SARS-CoV-2 variants have distinct activating properties of neutrophils

**Figure 2:**
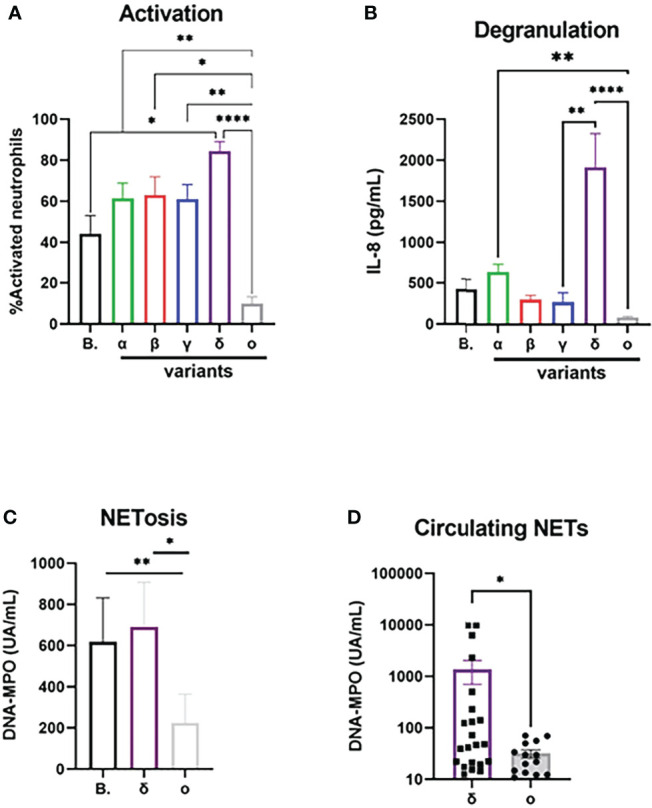
Activation potential of neutrophils differ between SARS-CoV-2 variants. **(A)** Percentage of CD11bhigh/CD62Llow activated neutrophils after 90 min incubation with SARS-CoV-2 “Wuhan” strain B and its major VOCs after normalization for medium (n=12). **(B)** Concentration of IL-8 in supernatant after 90 min incubation with SARS-CoV-2 after normalization for medium (n=12). **(C)** Concentration of DNA-MPO complexes in supernatant of isolated neutrophils after 3h of incubation with SARS-CoV-2 virus after normalization for medium (n=12). **(D)** Circulating concentration of DNA-MPO complexes in patients infected with Delta (n=22) or Omicron strain (n=14). Data are mean ± SEM, *p<0.05; **p<0.01; ****p<0.0001.

SARS-CoV-2 VOCs were cultivated from clinical isolates and used after infectious titration in neutrophil activation experiments in comparison with the original “Wuhan” (B) strain (**Figures 2A** **–C**). For a same MOI, incubation with the Delta variant induced a significantly higher percentage of neutrophil activation compared to the ancestral strain (84 ± 4% vs 44 ± 8%, p=0.03), while the Omicron variant induced a much lower activation than all other strains, in particular compared to the Delta strain (11.3 ± 3% vs 87 ± 4%, p<0.0001, **Figure 2A**). To confirm these results in a different model, we used lentiviral pseudotyped viruses expressing the spike protein from Delta and Omicron. In line with our results with the full virus, we found a significantly higher neutrophil activation with the Delta spike than with the Omicron spike (p<0.001; **Figure S3**). Next, we measured degranulated IL-8 in full virus-treated neutrophil supernatants, which showed an even larger difference for Delta variant compared to the Omicron variant (1914± 408 vs 87 ± 11 pg/mL, p<0.0001; **Figure 2B**). Since NETosis seems to be a prominent feature of neutrophil contribution to COVID-19 pathogeny, we tested NET generating capacity of SARS-CoV-2 variants circulating at the time of the study (Delta and Omicron) on isolated neutrophils. We show that the supernatants of neutrophils stimulated with the B. strain or the Delta strain contain higher NETs concentrations than those stimulated with the Omicron strain (618 ± 213 and 690 ± 217 vs 224 ± 139 UA/mL, p=0.0005 for both, **Figure 2C**). To confirm the impact *in vivo*, we measured circulating NET concentrations at diagnosis in patients infected with Delta or Omicron variants (**Table 1**), and found a higher concentration in patients with the Delta strain (1359 ± 658 *vs* 31.7 ± 5 UA/mL, p=0.027, **Figure 2D**) although blood neutrophil concentrations were similar in both groups (**Supplemental Figure 2**)

**Table 1:** Patients sera: These table contain age and gender of patients used to Netosis assay.

Delta sera			Omicron sera		
Patient	Age	Gender	Patient	Age	Gender
1	62	M	1	73	M
2	69	M	2	69	M
3	63	F	3	74	F
4	59	M	4	94	M
5	64	F	5	86	F
6	65	M	6	73	M
7	83	M	7	82	M
8	88	M	8	60	M
9	78	F	9	70	M
10	73	M	10	87	M
11	69	M	11	71	F
12	58	F	12	73	M
13	78	M	13	76	F
14	74	F	14	64	F
15	47	M	*Average age*		73
16	64	F	*Median age*		75
17	51	M	*Percentage of Men (%)*		64.3
18	64	M	*Percentage of Women (%)*		35.7
19	108	F
20	80	M
21	72	M
22	64	M
*Average age*		67
*Median age*		70
*Percentage of Men (%)*		68.2
*Percentage of Women (%)*		31.8

## Discussion

Neutrophils are major players in SARS-CoV-2 infection and neutrophil activation has been linked to severity and poor prognosis (12, 17, 18). Therefore, understanding neutrophil activation pathways in COVID-19 is of paramount importance to design neutrophil-targeted therapeutic interventions. In this study, we demonstrate that one of the neutrophil activation pathways could be direct activation by infectious viral particles. We show that, *in vitro*, human healthy resting neutrophils can sense SARS-CoV-2 by TLR7/8 and STING activation, upregulate membrane activation markers, degranulate IL-8 and produce ROS and NETs. Additionally, we show that SARS-Cov2 is able to delay neutrophil apoptosis *via* downregulation of active caspase 3 expression, which could participate to a sustained pro-inflammatory effect. We then compared the activation potential of neutrophil by SARS-CoV-2 variants and showed a significantly increased activation by the Delta variant compared to the ancestral strain and a decreased activation by the Omicron variant.

We and other have described early neutrophil activation in blood and tissue neutrophils from COVID-19 patients (12, 17, 18). This activation is mostly believed to be due to pro-inflammatory cytokine released in particular by macrophages and monocytes (19, 20). Indeed, plasma from severe patients can activate neutrophils from healthy donors (21–23). However, a study suggested this could be dependent on circulating immune complexes rather than cytokines (24). Here, we show that direct activation by the virus itself could contribute to neutrophil activation at infection sites. While a direct neutrophil activation would seem a counterproductive strategy for a virus, it has been shown that neutrophil proteases could participate in the maturation of the spike protein and thus facilitate infection (25, 26). More intriguing, it was recently suggested that the SARS-CoV-2 could use the histones on NETs as a hook to facilitate infection of neighboring cells *via* sialic acid binding (27).

The quantity of virus necessary to activate neutrophils seemed relatively large as compared to what is usually necessary to infect epithelial cells (28). However, at infection sites there is a huge amplification of viral particles due to viral replication. Moreover, we used untouched resting neutrophils from healthy donors that are less responsive to weak stimuli. Indeed, it is well described that neutrophil activation is a multistep process and that a priming step is necessary to get a full response (29). In an infectious context, neutrophils recruited to tissues are primed by the chemotaxis and diapedesis process and arrive in an inflammatory environment, making them likely to respond to much lower quantities of free viral particles.

Some studies have already shown activation of neutrophils by SARS-CoV-2 (8, 9), but none have compared the activation potential of all major variants so far. In doing so, we demonstrated a significant difference between Delta and Omicron variants, the latter being much less active on neutrophils. Interestingly, we were able to link this finding to clinical data since patients with Omicron presented less NETs in their serum, a fact recently confirmed by a preliminary study (30). Clinically, it is now well established that infection with the Omicron variant causes less severe disease that infection with Delta with a significantly lower hospitalization and lethality rate (31, 32). Since neutrophils are believed to be instrumental in disease severity, one may speculate this could participate to the less severe disease phenotype.

In addition, our results with spike-expressing pseudoviruses strongly suggests that the spike protein plays an important role in neutrophil activation. Indeed, it has been shown that the spike protein alone can induce neutrophil activation and NETosis (33). Additionally, one team has recently demonstrated that the Omicron spike protein presented a new cleavage site for neutrophil Cathepsin G protease (25), a feature which might interfere with its neutrophil activation properties.

The main limitation of this study is the *in vitro* experiments that do not reproduce accurately the inflamed setting in which the neutrophil meets the virus. However, this bias was necessary to be able to distinguish the signal induced by the virus itself from the one induced by the inflammatory background. In the future, studies on more elaborate models including infected epithelial cells will allow to explore further the virus-neutrophil interactions in a more physiological setting. Additionally, we did not explore the effect of co-infection with several VOCs. Indeed, co-infections with several VOCs have been described (34). While the clinical significance of such occurrence is not yet well known, in light of our data, it would be interesting to study their effect on innate immune response.

In conclusion, we demonstrate that neutrophil activation in COVID-19 patients can be done directly by the virus, and that the differences of clinical severity between the different strains of SARS-CoV-2 could be caused by differences in neutrophil activation potential. These data highlights even more the need to address neutrophil-virus interactions as an additional potential target for therapeutic intervention in SARS-CoV-2 infection.

## Data availability statement

The raw data supporting the conclusions of this article will be made available by the authors, without undue reservation.

## Ethics statement

The studies involving human participants were reviewed and approved by National Ethics committee Ile de France 8 n°2020-A02676-33. Written informed consent for participation was not required for this study in accordance with the national legislation and the institutional requirements.

## Author contributions

LDC and SL contributed to conception and design of the study. LDC and SL organized the database. SL and LDC wrote the first draft of the manuscript. All authors contributed to manuscript revision and read and approved the submitted version. OD and CR contributed to the technical implementation of the revisions. All authors contributed to the article and approved the submitted version.

## Funding

This study has been funded in part by grant #AC43 of the French “Agence Nationale de Recherche sur le SIDA et les hépatites virales” (ANRS).

## Acknowledgments

We wish to thank the team of the National Reference Center (CNR) of Mycobacteria for their valuable help in this work.

## Conflict of interest

The authors declare that the research was conducted in the absence of any commercial or financial relationships that could be construed as a potential conflict of interest.

## Publisher’s note

All claims expressed in this article are solely those of the authors and do not necessarily represent those of their affiliated organizations, or those of the publisher, the editors and the reviewers. Any product that may be evaluated in this article, or claim that may be made by its manufacturer, is not guaranteed or endorsed by the publisher.
