# Supplementary figures and images for: Differential activation of human neutrophils by SARS-CoV-2 variants of concern
Source: Front Immunol. 2022 Oct 27;13:1010140. doi: 10.3389/fimmu.2022.1010140 (PMC9646985; doi:10.3389/fimmu.2022.1010140)

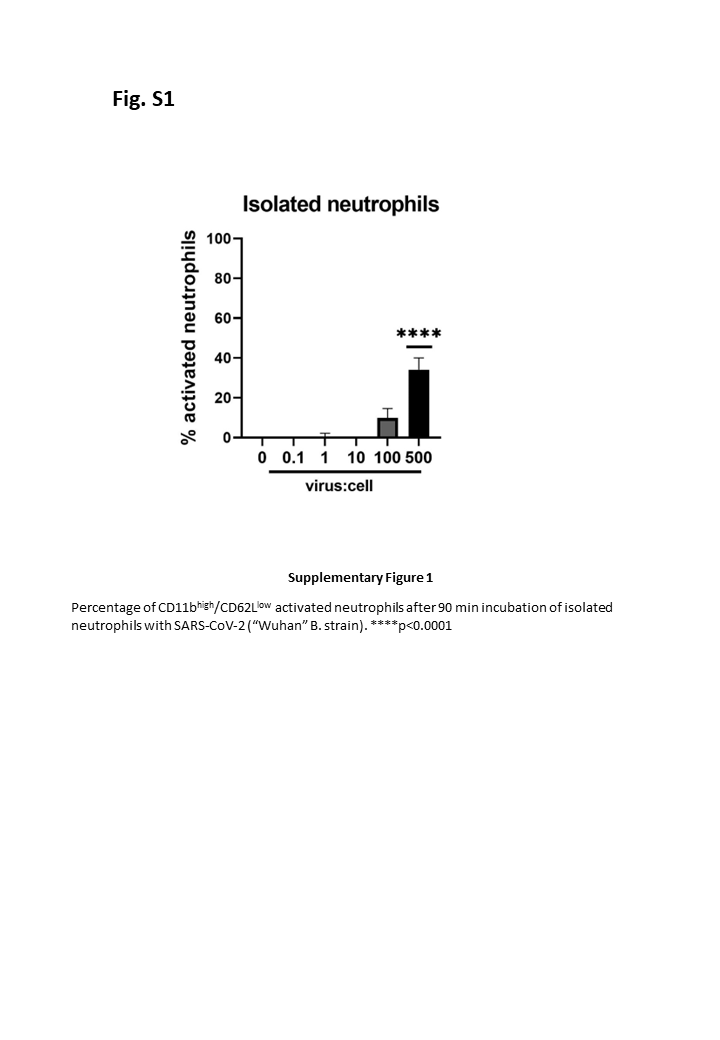

Supplement: Supplementary file 1 [file Image_1.tif]

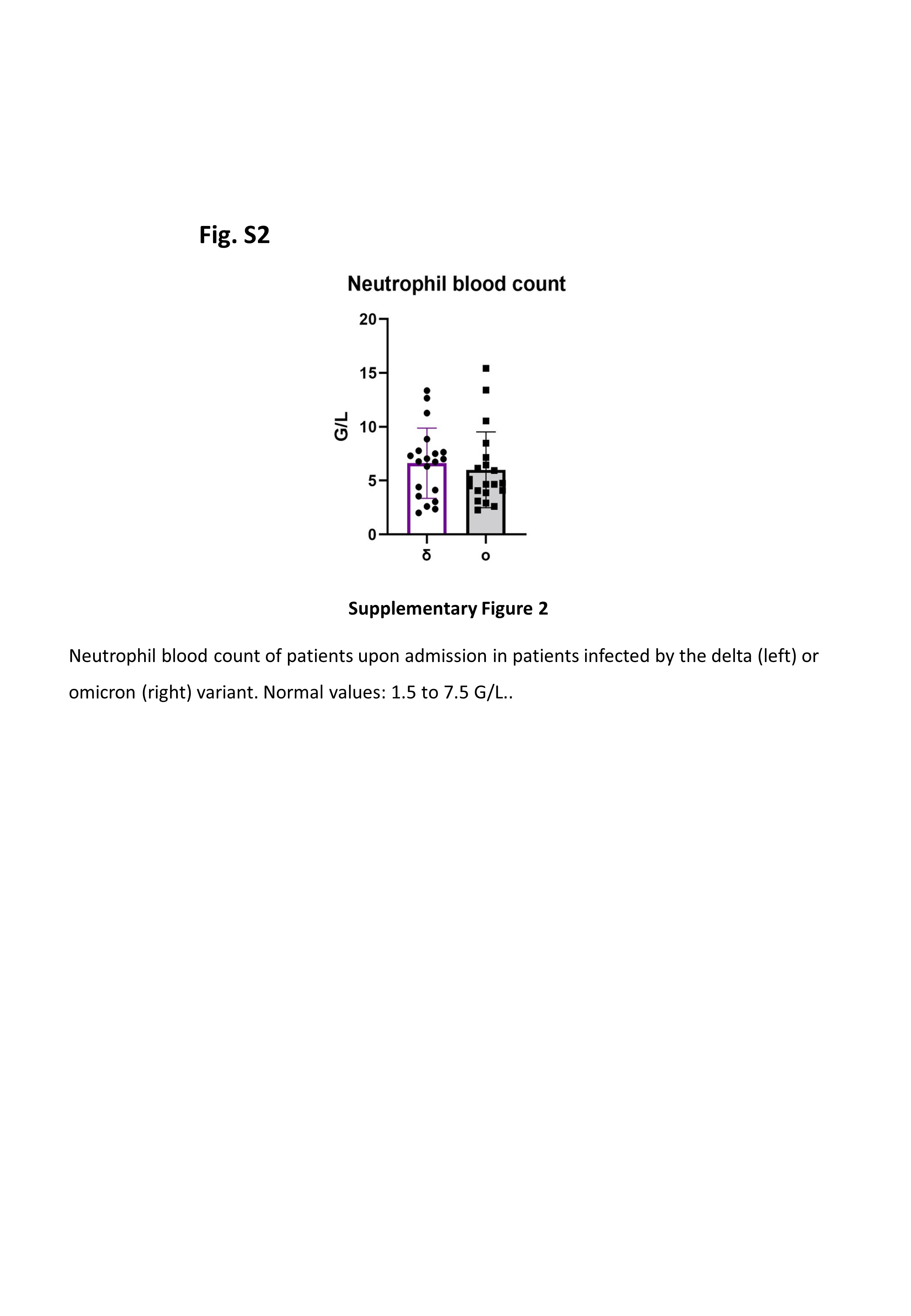

Supplement: Supplementary file 2 [file Image_2.tif]

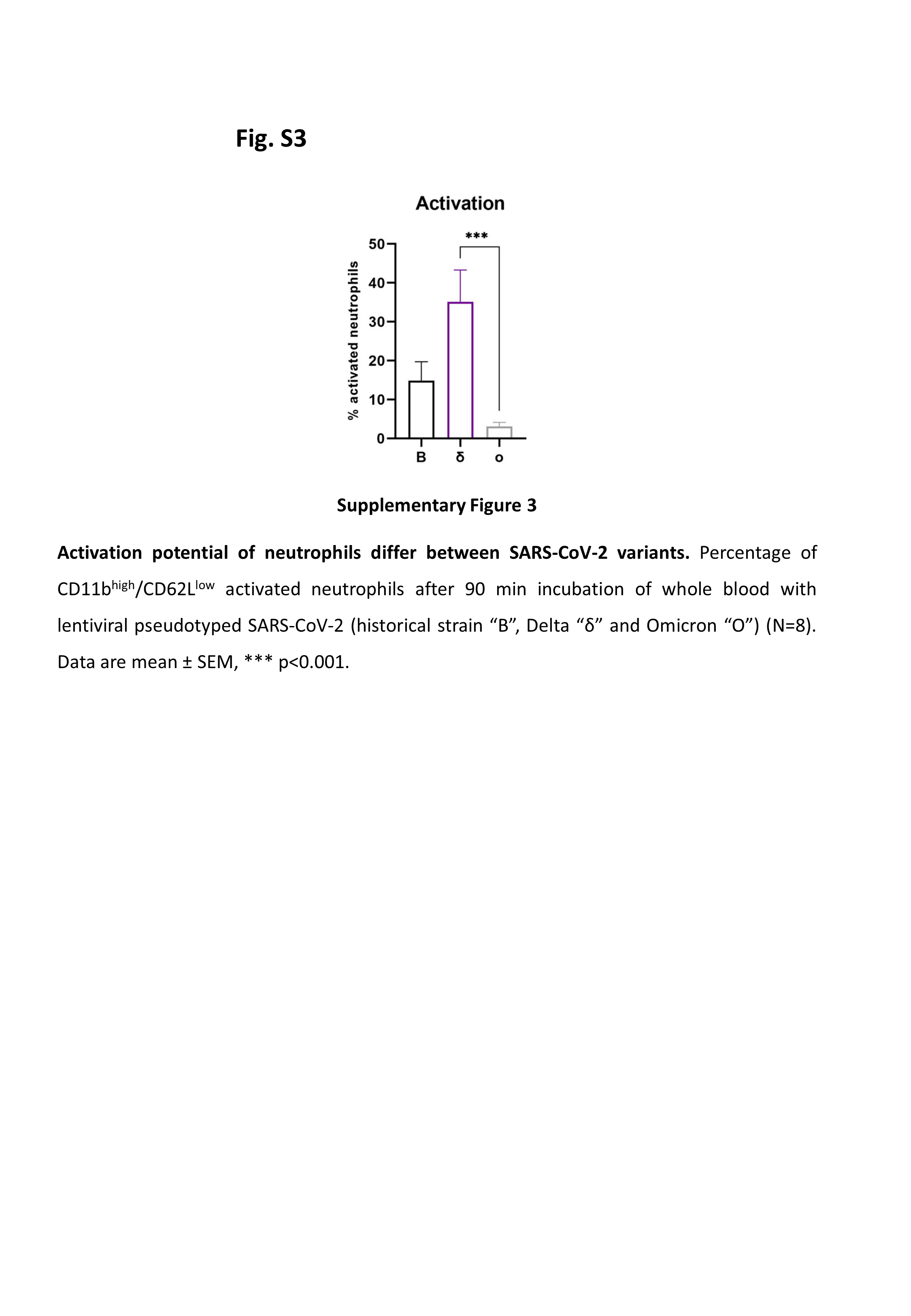

Supplement: Supplementary file 3 [file Image_3.tif]
